# The Reliability and Clinical Validation of Automatically-Derived Verbal Memory Features of the Verbal Learning Test in Early Diagnostics of Cognitive Impairment

**DOI:** 10.3233/JAD-230608

**Published:** 2024-01-02

**Authors:** Nina Possemis, Daphne ter Huurne, Leonie Banning, Angelique Gruters, Stephanie Van Asbroeck, Alexandra König, Nicklas Linz, Johannes Tröger, Kai Langel, Arjan Blokland, Jos Prickaerts, Marjolein de Vugt, Frans Verhey, Inez Ramakers

**Affiliations:** a Alzheimer Centre Limburg, School for Mental Health and Neuroscience, Maastricht University, Maastricht, The Netherlands; b Maastricht University Medical Centre+ (MUMC+), Department of Psychiatry & Psychology, Maastricht, The Netherlands; c Catharina Hospital, Medical Psychology, Eindhoven, The Netherlands; d National Institute for Research in Computer Science and Automation (INRIA), Valbonne, Sophia Antipolis, France; e ki:elements, Saarbrücken, Germany; f Janssen Clinical Innovation, Beerse, Belgium; g Faculty of Psychology and Neuroscience, Department of Neuropsychology & Psychopharmacology, EURON, Maastricht University, Maastricht, The Netherlands; h School for Mental Health and Neuroscience, Department of Psychiatry and Neuropsychology, Maastricht University, Maastricht, The Netherlands

**Keywords:** Alzheimer’s disease, cognitive dysfunction, memory, speech

## Abstract

**Background:**

Previous research has shown that verbal memory accurately measures cognitive decline in the early phases of neurocognitive impairment. Automatic speech recognition from the verbal learning task (VLT) can potentially be used to differentiate between people with and without cognitive impairment.

**Objective:**

Investigate whether automatic speech recognition (ASR) of the VLT is reliable and able to differentiate between subjective cognitive decline (SCD) and mild cognitive impairment (MCI).

**Methods:**

The VLT was recorded and processed via a mobile application. Following, verbal memory features were automatically extracted. The diagnostic performance of the automatically derived features was investigated by training machine learning classifiers to distinguish between participants with SCD versus MCI/dementia.

**Results:**

The ICC for inter-rater reliability between the clinical and automatically derived features was 0.87 for the total immediate recall and 0.94 for the delayed recall. The full model including the total immediate recall, delayed recall, recognition count, and the novel verbal memory features had an AUC of 0.79 for distinguishing between participants with SCD versus MCI/dementia. The ten best differentiating VLT features correlated low to moderate with other cognitive tests such as logical memory tasks, semantic verbal fluency, and executive functioning.

**Conclusions:**

The VLT with automatically derived verbal memory features showed in general high agreement with the clinical scoring and distinguished well between SCD and MCI/dementia participants. This might be of added value in screening for cognitive impairment.

## INTRODUCTION

Neurodegenerative diseases, such as Alzheimer’s disease (AD), develop gradually and in their early phase it is not often easy to distinguish from normal aging. In patients with very mild cognitive symptoms, it is difficult to predict the individual disease course and risk of progression to dementia. An acute and personalized diagnosis is critical to provide the appropriate care and guidance to people seeking help for their cognitive complaints [1].

Verbal memory tests, such as word-list recall tests, are widely endorsed tests for measuring verbal declarative memory impairment in the early diagnostics of cognitive impairment and dementia. An example of such a word-list recall test is the verbal learning test (VLT), a well-validated, reliable, and examiner-administered instrument widely used to measure verbal episodic memory. It has high sensitivity and specificity to distinguish participants with mild cognitive impairment (MCI) and dementia from controls and dementia from MCI [2, 3]. For example, Hamel and colleagues have shown that deterioration of memory performance on the VLT could be detected about 7 years before the dementia diagnosis [4]. Specifically, process scores such as serial position effects and semantic clustering have been shown to increase sensitivity and earlier detection of cognitively intact older adults at risk for cognitive decline and may reveal differences in performance between individuals with different subtypes ofMCI [5–7].

Computerized advancements in neuropsychological assessments offer more adaptive and sensitive measures for detecting cognitive impairment [8]. Therefore, they provide practical advantages of automated speech recognition (ASR) and reporting, such as ease of language adjustments, and reduced need for trained professionals, which in turn enable efficient and scalable administration for large-scale screening [9]. In general, existing literature has shown that complementary more fine-grained variables (e.g., speech breaks and semantic relatedness), rather than just clinical total scores, have been shown to aid automatic and early detection of cognitive impairment [10–13]. Notwithstanding, the clinical and diagnostic merit of the related and novel VLT features (such as total serial clusters, peak learning slope, and constancy learning index) remains to be explored. A meta-analysis of clinical total scores of the VLT has shown low to moderate correlation with other cognitive tests such as story recall tasks, the semantic verbal fluency (SVF) task, and the digit symbol substitution test (DSST) [14]. However, the relationship between automatically derived, more fine-grained VLT features and other cognitive tests, as well as disease severity remains unknown. The accuracy of automatically derived features in the early diagnostics of cognitive disorders, such as AD could provide more insight into verbal memory, thus leading to a non-invasive, cost-effective tool for diagnostics and prescreening in clinical trialdesigns.

In the present study, we investigated the accuracy of automated processing of the VLT compared to clinical scoring. Additionally, we were interested in the diagnostic accuracy and added value of automatically derived speech VLT features in clinical practice for distinguishing people with subjective cognitive decline (SCD) versus MCI and dementia compared to the gold standard, which is represented by the clinical VLT-total scores (total immediate recall, delayed recall and recognition count) used in clinical practice. Lastly, we investigated the relationship between these automatically derived VLT verbal memory features and other cognitive tasks, as well as disease severity, and functioning in daily living.

## METHODS

### Participants

As part of the DeepSpA (Deep Speech Analysis) project, 138 participants from the BioBank Alzheimer Centre Limburg (BBACL) study were included between 2019 and 2021 (Table 1). The BBACL study is an ongoing prospective cohort study that includes patients who were all referred to the memory clinic of the Maastricht University Medical Center+ (MUMC+). Of these 138 participants, 69 were diagnosed with SCD, and 69 were diagnosed with MCI or dementia (56 with MCI and 13 with mild dementia). Out of 138 participants, 137 had an MRI/CT scan available (including measures of medial temporal lobe atrophy (MTA), white matter abnormalities (WMA, i.e., Fazekas), and global cortical atrophy (GCA)). Inclusion criteria were a total score of ≥20 on the Mini-Mental State Examination (MMSE) [15, 16] and Clinical Dementia Rating scale (CDR) [17] global score of ≤1. Exclusion criteria were non-degenerative neurological diseases, a recent history of severe psychiatric disorders, the absence of a reliable informant, and the clinical judgment that a follow-up assessment after one year would not be feasible. Experiments on human subjects were performed in accordance with the ethical standards of the Committee on Human Experimentation of our Institution, which is in accordance with the Helsinki Declaration of 1975. The local Medical Ethical Committee (METC azM/UM) approved the study (MEC 15-4-100). Each participant had given their written informed consent before theassessments.

**Table 1:** Participant characteristics and group comparisons between individuals with and without cognitive impairment

	SCD	MCI/dementia	*p*
	(N = 69)	(N = 69)
Age, y	62.4 (10.8)	71.9 (9.5)	<0.001
Sex (% men)	65.7	58.8	0.404
Education (% low, mid, high)	27.1/37.1/35.7	39.7/30.9/29.4	0.496
CDR SOB	0.8 (0.9)	2.0 (1.7)	<0.001
DAD (total %)	94.7 (7.9)	83.6 (15.9)	<0.001
MMSE	28.7 (1.2)	26.1 (2.6)	<0.001
GDS-15	3.1 (2.3)	3.2 (2.9)	0.883
15-VLT total immediate recall (ASR)	37.6 (12.9)	24.1 (10.7)	<0.001
VLT delayed recall (ASR)	8.1 (3.5)	3.6 (3.2)	<0.001
Z-score SVF	–0.8 (0.9)	–1.2 (0.9)	<0.001
Z-score Stroop-III	0.0 (1.1)	–1.7 (3.8)	0.001
Z-score TMT-B or CST-C	0.1 (1.1)	–1.2 (2.1)	<0.001
Z-score RBMT immediate recall	–0.3 (1.0)	–1.1 (0.9)	<0.001
Z-score RBMT delayed recall	–0.2 (1.0)	–1.2 (1.0)	<0.001

Data are presented as mean (SD) unless otherwise specified. MMSE, Mini-Mental State Examination; DAD, Disability Assessment for dementia; CDR SOB, Clinical Dementia Rating scale Sum of Scores; GDS-15, Geriatric Depression Scale-15 items; 15-VLT, 15-Word Verbal Learning Test; SVF, Semantic Verbal Fluency; TMT, Trail Making Test; CST-C, Concept Shifting Test; RBMT, Rivermead Behavioral Memory Test; SCD, subjective cognitive decline; MCI, mild cognitive impairment; ASR, automatic speech recognition.

### Clinical assessment

Each participant underwent a standardized assessment including a medical history taking, a neurological and psychiatric assessment, and several questionnaires to measure disease severity (CDR), functioning in daily living (Disability Assessment for Dementia, DAD) [18, 19], and depressive symptomatology (Geriatric Depression Scale-15 items, GDS-15) [20]. In addition, participants underwent an extensive neuropsychological assessment, consisting of a test for measuring global cognition (MMSE), episodic memory (15-word Verbal Learning Test) [21, 22], & Storytelling of the RBMT (Rivermead Behavioral Memory Test), semantic memory (Semantic Verbal Fluency, SVF) [23], attention and executive functioning (Concept Shift Test, CST) [23] (or if not available the Trail Making Test [24], and Stroop [25]). The multidisciplinary clinical diagnosis was based on the Diagnostic and Statistical Manual of Mental Disorder (DSM-IV-TR, DSM-5) criteria for MCI (cognitive disorder not otherwise specified (NOS) in DSM-IV-TR; mild neurocognitive disorder in DSM-5; and dementia (DSM-IV) or major neurocognitive disorder (DSM 5)) [26, 27]. AD dementia diagnoses were made according to McKhann’s [28] Core Clinical criteria, meaning that patients diagnosed with AD implicated an amnestic memory profile, insidious onset, and history of deterioration of cognition by report or observation [28]. When cognitive impairments could not be objectified, participants were classified as having SCD [29].

### Word-Verbal Learning Test (15-VLT)

This study used the Dutch 15-word Verbal Learning Test (VLT) [22], which is an adaptation of the commonly used Rey Auditory Verbal Learning Test (RAVLT) [30]. Trained clinical psychodiagnostic test leaders presented (visual stimulus presentation) 15 unrelated monosyllabic nouns. After this presentation, the participants had to recall each word they remembered. The VLT consists of five learning trials, resulting in the total number of correctly remembered words (total immediate recall). After 20 min of nonverbal and non-memory tasks, individuals were (unexpectedly) asked again to recall all words they could remember (delayed recall). Finally, a list of 30 words was presented in which the 15 stimulus words were intermixed with 15 nontarget words and the participant had to recognize the words from the stimulus list (recognition) [21]. Three parallel list versions of the Dutch 15-VLT were used. Clinical scores include total immediate recall (sum of trial 1 to trial 5), delayed recall, and recognition count (true positive).

### Speech data recording and processing

The VLT was audio recorded, scored, and processed using a mobile application provided by ki:elements GmbH (iOS iPad version; ki:elements, 2022). The application recorded participants’ speech responses while they performed neuropsychological assessments in the clinic. The application used the iPad’s standard internal microphone, which was placed in front of the participant. After speech responses were recorded, they were sent to the backend of ki:elements for preprocessing (such as cutting recordings into relevant parts and audio transformation), automatic speech recognition, and feature extraction [31]. This resulted in two different measurements of both the total immediate recall and delayed recall: the automatically derived ASR score, and the clinician’s independent score. Based on the automatically derived application scores, 102 VLT-specific performance metrics such as serial-positioning effects, slopes, and subjective organization & serial clustering were automatically calculated. Note that the clinical recognition count was manually added to the application. See Supplementary Table 1 for a complete listing of the VLT features.

### Statistical analyses

The data were analyzed using IBM SPSS Statistics Mac (version 27) and R 4.1.2 (R Core Team, 2021). Group differences were analyzed with independent *t*-tests for continuous variables and with Chi-square tests for categorical variables. When a variable was not normally distributed a Mann-Whitney-U test was performed. Educational level was categorized into low (at most primary education), mid (junior vocational training), and high (senior vocational or academic training) according to a Dutch grading system [32], which is comparable with the Standard Classification of Education [33]. The intraclass correlation coefficient (ICC) of the total scores was calculated to examine the agreement between the ASR-based total immediate recall and delayed recall score and the independent clinical total immediate recall and delayed recall score, based on a mean-rating (k = 2), absolute-agreement, 2-way-mixed-effects model. Effect sizes for the verbal memory features were calculated using the Z-statistic of the Mann-Whitney U test (|Z|/√N), a non-parametric test due to the skewness of most of the verbal memory features. To visualize correlations between features and other cognitive tests, a correlation matrix in form of a heatmap was constructed using the R software package (version 3.6) and the package corrplot. Age, sex, and education-adjusted Z-scores of the cognitive tests were based on published normative data for the Dutch population [23]. Correlation strength was interpreted based on Akoglu [34].

In Python 3.9.7, machine learning models (Extra Trees classifier) were trained to differentiate between the two different groups (SCD versus MCI/dementia) using the sklearn Python package [35]. Extra Trees is an ensemble tree-based machine-learning approach. Due to the limited sample size, no held-out test set could be maintained. Instead, models were evaluated using Leave-One-Out Cross-Validation, a procedure in which one sample at a time is removed from the training set and used as a test case. This procedure was repeated for each sample and the average of the model’s performance was calculated. The area under the receiver operating characteristics curve (AUC-ROC), which allows visualization of multiple different potential trade-offs between sensitivity and specificity, was created for three models (model 1 crude: VLT-ASR total score; model 2: model 1 and age, model 3: model 2 and ASR based verbal memory features) for each VLT subtest separately, as well as the full VLT (total immediate recall, delayed recall, and recognition count). Confidence intervals (CI), *p*-values (DeLong method), and F1-scores for all AUC-ROCs were calculated using the sklearn Python package.

## RESULTS

### Sociodemographic and clinical data

Demographic information of participants with SCD and MCI/dementia are presented in Table 1. As expected, the MCI/dementia group was older than the SCD group and had lower performances on all cognitive tests and a higher CDR-Sum of Boxes score. No significant group differences were found for sex, education level, and GDS-15 score. The most common etiology of the MCI/dementia group was AD (48%). Residual etiologies included vascular etiology (30%), mixed (AD and vascular) etiology (6%), and other non-cognitive disorders (16%) such as MCI due to Parkinson’s disease. There were no clear signs of evident medial temporal lobe atrophy in 93% of the SCD group.

### Inter-rater reliability between automatic and manual scoring

**Fig. 1:**
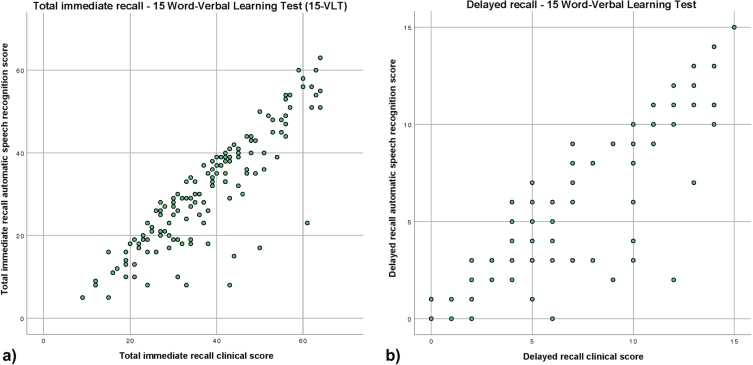
Scatterplots of the clinical score and automatic speech recognition of the VLT (a) total immediate recall, (b) delayed recall.

The ICC for the inter-rater reliability between the clinical score and the ASR of the total immediate recall was 0.87 (95% CI 0.28–0.95; Fig. 1a). The mean difference between the clinical score and the ASR was 7 words with a range from –1 to 38 words. Except in one case, the ASR detected fewer words than the clinical score. In 13 out of 138 (9.5%) people, the ASR missed more than 14 words. A sensitivity analysis showed that the ICC was lower for the SCD group (0.77 95% CI 0.09–0.91) than for the MCI/dementia group (0.88 95% CI 0.10–0.96). When separating the MCI/dementia group in participants with MCI and participants with dementia, results showed that the ICC was better in participants with MCI (0.87 95% CI 0.08–0.96) than in participants with dementia (0.82 95% CI –0.15–0.96).

The ICC for the inter-rater reliability between the clinical score and the ASR of the delayed recall of the VLT was 0.94 (95% CI 0.88–0.97; Fig. 1b). The mean difference between the clinical score and the ASR scoring was 1 word with a range from –2 to 10 words. In general, the ASR detected fewer words than the clinical score. In 7 out of 138 (5%) people, the ASR missed more than 4 words.

A sensitivity analysis showed that the ICC was lower for the SCD group (0.86 95% CI 0.64–0.93) than for the MCI/dementia group (0.95 95% CI 0.91–0.97). When separating the MCI/dementia group in participants with MCI and participants with dementia, results showed that the ICC was comparable in both groups (MCI; 0.94 95% CI 0.89–0.97, dementia; 0.95 95% CI 0.85–0.98).

In a posthoc analysis, we analyzed how many words were mentioned in the first 10 s of each of the 5 immediate trials. We found that about half of the recalled words were mentioned in the first 10 s. Looking at the ICC for each immediate recall trial and group individually, we saw that the ICCs per trial for the group with MCI/dementia stayed quite stable compared to the group with SCD, for which trial 1 starts with a high ICC but declines to a lower ICC for all the residual trials (See Supplementary Table 2 for posthoc results).

### Diagnostic classification

**Fig. 2:**
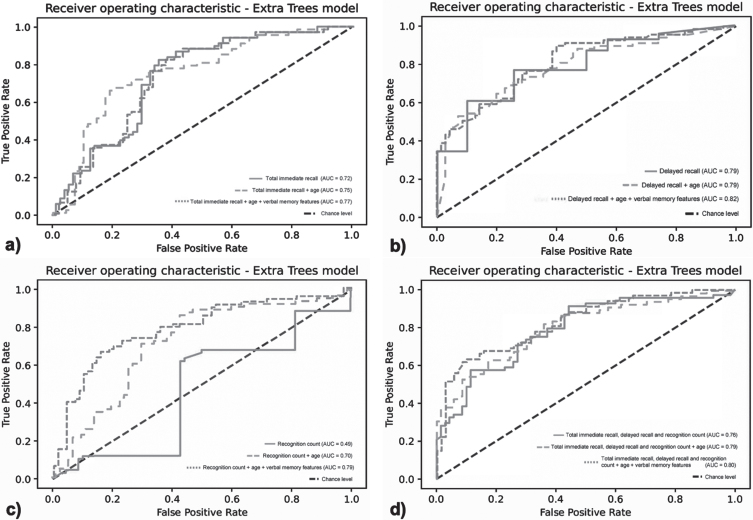
Receiver operator curves of the extra trees classification model for classifying SCD versus MCI/dementia. a) Model 1: total immediate recall only; model 2: total immediate recall and age; model 3: total immediate recall, age, and verbal memory features, b) Model 1: delayed recall only; model 2: delayed recall and age; model 3: delayed recall, age, and verbal memory features, c) Model 1: recognition count only; model 2: recognition count and age; model 3: recognition count, age, and verbal memory features, d) Model 1: total immediate recall, delayed recall, and recognition count only; model 2: total immediate recall, delayed recall, recognition count and age; model 3: total immediate recall, delayed recall and recognition count, age, and verbal memory features.

The ROC curves differentiation between the SCD group and MCI/dementia group for the total immediate recall is shown in Fig. 2a. The full model including the total immediate recall, age, and verbal memory features (model 3) was able to differentiate between the SCD group and the MCI/dementia group (AUC = 0.77, 95% CI 0.70–0.85, F1-score = 0.65). The full model including the verbal memory features had a slightly higher AUC compared to the age-corrected total immediate recall (model 2) (AUC = 0.75, 95% CI 0.68–0.84, F1-score = 0.74) and the total immediate recall only (model 1) (AUC = 0.72, 95% CI 0.64–0.81, F1-score = 0.73). When comparing whether the models differ from each other, no significant differences can be found.

Figure 2b shows the differentiation between the SCD group and MCI/dementia group for delayed recall. The full model including the delayed recall, age, and verbal memory features was able to differentiate between both groups (AUC = 0.82, 95% CI 0.75–0.89, F1-score of 0.70). The full model (model 3) including the verbal memory features had a slightly higher AUC compared to the age-corrected delayed recall (model 2) (AUC = 0.79, 95% CI 0.71–0.87, F1-score of 0.71) and the delayed recall only (model 1) (AUC = 0.79, 95% CI 0.71–0.86, F1-score of 0.65). When comparing whether the models differ from each other, no significant differences canbe found.

Figure 2c shows the differentiation between the SCD group and the MCI/dementia group for the recognition count. The full model including the recognition count, age, and verbal memory features (model 3) was able to differentiate between both groups (AUC = 0.79, 95% CI 0.72–0.88, F1-score of 0.72). The full model including the speech features had a substantially higher AUC compared to the age-corrected recognition count (model 2) (AUC = 0.70, 95% CI 0.61–0.79, F1-score of 0.69) and recognition count only (model 1) (AUC = 0.47, 95% CI 0.38–0.58, F1-score of 0.61). When comparing whether the models differ from each other, significant differences can be found for all models (model 1 versus model 2; *p* < 0.001, model 1 versus model 3; *p* < 0.001, and model 2 versus model 3; *p* < 0.01).

Figure 2d shows the differentiation between the SCD group and the MCI/dementia group for the full VLT. The full model including the total immediate recall and delayed recall, recognition count, age, and verbal memory features (model 3) was able to differentiate between both groups (AUC = 0.80, 95% CI 0.73–0.87, F1-score of 0.72). The full model including the verbal memory features had a slightly higher AUC compared to the age-corrected model (model 2) (AUC = 0.79, 95% CI 0.72–0.87, F1-score of 0.71) and the model with the clinical score only (model 1) (AUC = 0.76, 95% CI 0.67–0.84, F1-score of 0.68). When comparing whether the models differ from each other, no significant differences can be found.

A sensitivity analysis showed that after excluding the participants with dementia (N = 13), the AUC of total immediate recall decreased slightly from 0.68 (model 1) to 0.67 (model 3). For delayed recall, the AUC increased from 0.72 to 0.77. For recognition count, the AUC increased from 0.39 to 0.72. Lastly, for the full model including total immediate recall, delayed recall, and recognition count the AUC increased from 0.73 to 0.79. In comparison to the results of the analysis including patients with dementia, the crude models were slightly lower when patients with dementia wereexcluded.

### Effect sizes of the automatically derived verbal memory features and discriminative power

Table 2 shows the 10 features with the best discriminative ability of all ASR-based 102 VLT features, including total scores, between the SCD group and MCI/dementia group (see Supplementary Table 1 for a more detailed description of the features). The highest effect sizes were found for 1) delayed recall, 2) midlist item counts trial 3, 3) delayed recall midlist items, 4) late learning slope, 5) immediate total midlist items, 6) immediate count trial 5, 7) total immediate recall, 8) immediate count trial 4, 9) delayed recall recency items, 10) immediate count trial 3. A sensitivity analysis, in which participants with dementia were excluded from the cognitively impaired group, resulted in the same 10 best differentiating features, with a different ascending order only.

**Table 2:** Best 10 ASR-based features to differentiate participants with SCD and MCI/dementia

Feature	Z-value	*p*	Effect size	AUC score
Delayed recall	–6.824	<0.001	0.581	0.79
Immediate midlist items trial 3	–5.991	<0.001	0.510	0.72
Delayed recall midlist items	–5.990	<0.001	0.510	0.76
Late learning slope	–5.988	<0.001	0.510	0.72
Immediate total midlist items	–5.943	<0.001	0.506	0.65
Immediate count trial 5	–5.935	<0.001	0.505	0.74
Total immediate recall	–5.817	<0.001	0.500	0.73
Immediate count trial 4	–5.544	<0.001	0.471	0.68
Delayed recall recency items	–5.538	<0.001	0.471	0.66
Immediate count trial 3	–5.522	<0.001	0.470	0.69

### Correlations between the 10 best ASR VLT features and other cognitive tests

**Fig. 3:**
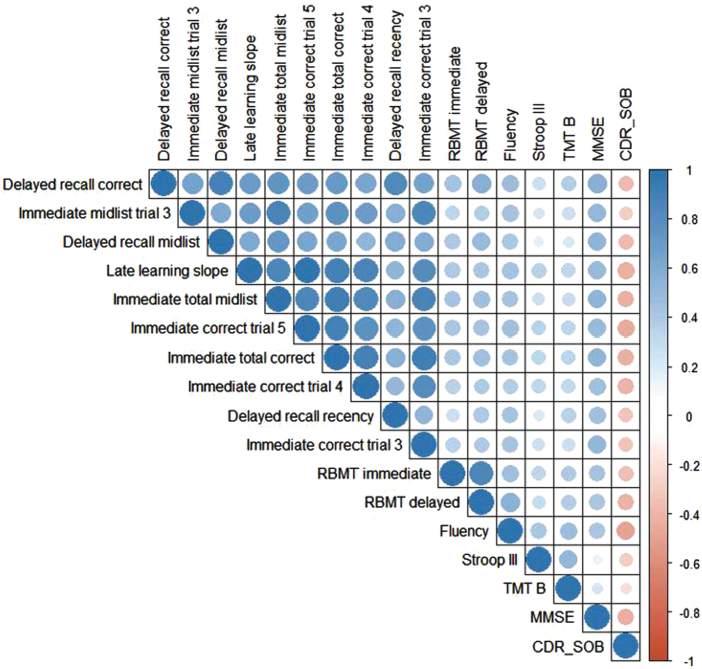
Heatmap for observed Pearson correlation coefficients between the automatically retrieved VLT features, cognitive tests, and daily functioning. Positive correlations are presented in blue and negative correlations are presented in red. A stronger correlation is represented by a darker color and a bigger circle. N = 99 due to case-wise deletion in case of missing values. RBMT, Rivermead Behavioral Memory Test; TMT-B, Trail Making Test B; MMSE, Mini-Mental State Examination; CDR-SOB, Clinical Dementia Rating scale Sum of Scores.

Of 138 participants, 99 had all cognitive test performances available. All 10 VLT features were significantly correlated with each other, ranging from a moderate correlation *r*(136) = 0.57, *p* < 0.01 (immediate count trial 4 and delayed recall midlist items) to a very strong correlation *r*(136) = 0.92, *p* < 0.01 (immediate count trial 3 and immediate total) (Fig. 3). Regarding cognitive functioning in general, all 10 VLT features were significantly positively correlated with the MMSE ranging from a moderate correlation *r*(135) = 0.46, *p* < 0.01 (delayed recall recency) to a moderate correlation *r*(135) = 0.56, *p* < 0.01 (total immediate recall). All 10 VLT features were significantly positively correlated with the semantic verbal fluency ranging from *r*(133) = 0.38, *p* < 0.01 (immediate count trial 4) to *r*(133) = 0.48, *p* < 0.01 (delayed recall). Regarding executive functioning, none of the VLT features were correlated to the Stroop-III, and only weak correlations were found between the VLT features and the TMT-B. For story recall, all 10 VLT features were significantly positively correlated to the RBMT delayed *r*(117) = 0.30, *p* < 0.01(immediate midlist trial 3) to *r*(117) = 0.50, *p* < 0.01 (delayed recall). The 10 VLT features were positively correlated to the immediate recall score of the RBMT. Concerning disease severity, all 10 VLT features had a significantly weak to moderate negative correlation with the CDR-SOB ranging from *r*(129) = –0.35, *p* < 0.01 (immediate midlist trial 3) to *r*(129) = –0.44, *p* < 0.01 (delayed recall).

## DISCUSSION

The current study evaluated the reliability and clinical validity of ASR technology compared with the clinical scoring of the 15-VLT in a memory clinic setting. Our results show that the ASR of the commonly used total immediate recall and delayed recall were comparable to the clinical retrieved scores with good reliability (based on [36]) for total immediate recall and excellent reliability for delayed recall. The higher ICC of the delayed recall can be explained by the difference in range between both measures (total immediate recall 0–75 versus delayed recall 0–15 words), resulting in a lower probability of error in the delayed recall. Note that we have to take the wide confidence intervals into account which indicates that further investigation is warranted and that current results should be optimized for individual diagnostic purposes. Our results showed that in 9.5% of participants, the ASR missed more than 14 words in the total immediate recall, and in 5% of participants, the ASR missed more than 4 words in the delayed recall. Looking into those cases in more detail, we determined that words were missed by the ASR when participants recalled words very quickly and without pauses between words, or spoke very quietly. In a posthoc analysis, we analyzed how many words were mentioned in the first 10 seconds of each of the 5 immediate trials. We found that about half of the recalled words were mentioned in the first 10 seconds. Interestingly, looking at the ICC for each immediate recall trial and group individually, we saw that the ICCs per trial for the group with MCI/dementia stayed quite stable compared to the group with SCD, for which trial 1 starts with a high ICC but declines to a lower ICC for all the residual trials (See Supplementary Table 2 for *post-hoc* results). Thus, by increasing the total number of recalled words with each trial and listing them quickly after the start of the recall phase, we see that the ICC decreases with each subsequent trial. In general, this could indicate that the ICC depends on the number of words recalled: the higher the word count the lower the ICC. Accordingly, we reason that participants who are instructed to recall words find it easier to recall them as quickly as possible so as not to forget words, possibly resulting in mumbling and recalling words without pausing in between. Additionally, some individuals might enumerate some words not intended to increase their total word count, but rather to invoice the order of words they learned, i.e., a learning efficiency strategy related to attention and learning [6]. The ASR might see these as repetitions when in reality they were not. In general, we expected that these effects interfere more with the ASR in the SCD group, which is confirmed by a lower ICC for the SCD group compared to the MCI/dementia group. However, these noise effects may be less applicable to delayed recall. Delayed recall is characterized as a measure of consolidation, thus reducing immediate recall strategies. To improve and mitigate such limitations of the ASR technology in the future, recall instructions should be optimized to emphasize that participants should speak clearly and separate recall words with small pauses in-between. Note that this in itself might be challenging by interfering with recall strategies, especially in individuals with cognitive impairment.

In the evaluation of the added value and accuracy of the additional automatically derived verbal memory features, we found that the full model including the total immediate recall, age, and verbal memory features was able to accurately distinguish between the SCD and the MCI/dementia group. The discriminative value of the full model is slightly higher than the model based on total immediate recall only. This reflects a slight increase in differentiating value of these automatically derived verbal memory features compared to the traditionally used clinical score. This suggests a 77% chance that the ASR of the 15-VLT and its verbal memory features can distinguish between both diagnostic groups, compared to a 72% chance with total immediate correct recall only [37]. The full model including delayed recall, age, and automatically derived verbal memory features were also able to accurately distinguish between the SCD group and the MCI/dementia group. This suggests an 82% chance that the ASR of the VLT and its verbal memory features can distinguish between both diagnostic groups, compared to a 79% chance with delayed recall only. Interestingly, there was a notable increase in the discriminative power of the prediction models regarding VLT recognition features. The full model including the recognition count, age, and the automatically derived features was able to accurately distinguish between the SCD group and the MCI/dementia group with a good discrimination ability. The AUC increased from a 47% chance with recognition count only, which is deemed unacceptable and similar to the chance level, to a 79% chance. When analyzing the ROC of the three commonly clinically used scores total immediate recall, delayed recall, and recognition count together, we found a discrimination ability of 76%. Adding all verbal memory features resulted in a 4% increase. Note that these results need to be interpreted carefully, as the increase was not statistically significant for all models, except the recognition sub-task, and the clinical value therefore questionable. Looking at the F1-scores of all models, all models were deemed acceptable, the lowest being 65% for model 3 from the immediate recall and model 1 from the delayed recall to the highest, 73% for model 2 of the immediate recall. Interestingly, model 3 of the immediate recall, which refers to the clinical score plus age correction and features, resulted in a lower F1-score than model 1, the clinical score alone. This was not applicable for the delayed recall, recognition, and all sub-tasks combined. In general, adding the verbal memory features increases discrimination ability in all separate subtasks and when performing the whole task including all subtasks. The increase in the AUCs cannot be explained by the age differences between groups because age was included in the second model for all AUCs. Accordingly, the results from this study suggest that automatic processing of the 15-VLT provides additional information beyond a clinician-rated total word count alone with no additional effort required. The sensitivity analysis excluding participants with dementia from the cognitively impaired group showed that participants with SCD and MCI could be differentiated, indicating that the 15-VLT is sensitive enough to detect differences in the early stages of cognitive deterioration.

Out of the 102 total additional automatically derived verbal memory features, we were interested in which ones differentiate most between participants with SCD and MCI/dementia. The best differentiating features were delayed recall, immediate midlist items trial 3, delayed recall midlist items, late learning slope, and the immediate total midlist items. Previous research has already demonstrated the diagnostic accuracy and sensitivity/specificity of good to excellent list learning tests were related to delayed recall and total immediate recall [3, 38, 39], and recognition count having poorer diagnostic accuracy than immediate or delayed recall scores [38]. Our results are in line with these findings and add value to the discriminative power of ASR-retrieved verbal memory features. The 10 best differentiating features were moderately to strongly correlated with each other. This is not unexpected, as the features represent different sub-parts of the VLT, all of which measure episodic memory. The highest correlation was seen between the immediate correct count in trial 3 and the total immediate recall, which could indicate a ceiling effect of the VLT after trial 3. Although ceiling effects are more common in a younger population, a study by Davis et al. [40] showed that when only 3 trials were administered, the age-related decline in the delayed recall and recognition test was comparable to administering 5 trials and thus reduced ceiling effects [40].

15-VLT features correlated with measures for other cognitive measures. The total immediate recall correlated moderately with the MMSE score. Although the MMSE measures global cognitive functioning, it includes an immediate one-trial 3-word list recall, delayed recall, and orientation in time and place and thus includes subtests measuring episodic memory which could explain the moderate correlation [41]. Correlations between the 15-VLT features and other memory tests such as the RBMT and the SVF resulted in low to moderate associations. These results are in line with a previous study which also found low to moderate correlations between the VLT and RMBT or SVF [14]. Looking at executive function, we did not find any significant correlations for the VLT features and inhibition effects (Stroop III), and only very weak correlations for the VLT and mental switching (TMT-B). Interestingly, these results are in line with Abulafia et al. [42] and Magalhães, Mallow-Diniz & Hamdan [43], who also did not find significant correlations between the VLT and the Stroop III or TMT-B [42, 43]. This confirms that the VLT indeed measures cognitive domains other than the Stroop III and TMT-B, i.e., executive function. The exact association between executive function and memory performance needs further attention as other studies found evidence for this relation [44]. Lastly, all best distinguishing features were negatively correlated to the CDR-SOB, i.e., disease severity, with the highest correlation for delayed recall. This might be caused by the relative weight of the memory domain included in the CDR. Accordingly, this possibly implies and confirms that the more severe the disease severity is, the lower the delayed recall is. Taken together, especially the delayed recall, including its verbal memory features, offers a high predictive diagnostic ability to distinguish cognitive impairment. In general, more research is needed in regard to the range in which the measures of the VLT and its automatically derived features correlate with other cognitive tests. Our current study was conducted in a face-to-face assessment at a memory clinic setting by recording and automatically processing the VLT. This speech-based analysis offers opportunities for remote neuropsychological testing [45]. Accordingly, it would be of great interest to investigate whether the VLT could be administered and processed remotely, e.g., by phone or video speech/conferencing platforms, to facilitate screening or participation of clinical trial participants or to monitor disease progression, e.g., for people living in medical deserts with less access to care facilities or patients who do not want to travel due to health precautions or limited mobility. Remote neuropsychological testing adapted to this specific population could have benefits in the future such as reduced (travel) costs and increased flexibility and comfort.

This study also has some limitations. This study was performed in a Dutch memory clinic setting, which in this case means that all participants were Caucasian and Dutch-speaking. Accordingly, findings cannot be generalized to the (healthy) general population. Further, this study consists of a relatively small sample. We had significant differences in age between groups, which is in line with other clinical studies, however, future studies could make use of an age-matched control design to overcome this limitation. As participants were recruited via the memory clinic of the MUMC+, Dutch clinical guidelines for the diagnostics of cognitive impairment and dementia were followed [46]. Accordingly, no PET scans are available, and cerebrospinal fluid is only available in a limited subset (N = 6). Further, we did not use a hold-out validation set, i.e., models were not naive to classification. Although this is state of the art and performs good validation for machine-learning models, we suggest that in the future, an independent validation cohort from another study would be interesting to check robustness between studies and cohorts. Additionally, intrusions could not be identified by the ASR technology. Intrusions refer to the participants recalling words that were not on the list, i.e., inaccurate memory. In general, susceptibility to intrusion effects has been associated with a higher probability of underlying cognitive decline at the prodromal phase of MCI [47, 48]. Thus, improving language detection in ASR to discern intrusions would be beneficial for future ASR studies that include the VLT as a measure of verbal memory.

In conclusion, the VLT and its associated ASR-derived verbal memory features can distinguish participants with SCD from those with MCI and dementia. Current results present ASR scores that are close to being consistent with clinical scores regarding discrimination ability in diagnosing cognitive impairment. Thus, the ASR and associated verbal memory features of the VLT could be potentially used in clinical diagnostics or to facilitate a non-invasive tool to screen participants. An integrated approach of ASR in semi or fully-automated (telephone) assessments might improve efficiency and accelerate recruitment in clinical trials, as no clinician would be needed to score the neuropsychological tests.

## Supplementary Material

Supplementary Material

## Data Availability

The data supporting the findings of this study are available on request from the corresponding author. The data are not publicly available due to privacy or ethical restrictions.
