# Supplementary material for: The Reliability and Clinical Validation of Automatically-Derived Verbal Memory Features of the Verbal Learning Test in Early Diagnostics of Cognitive Impairment
Source: J Alzheimers Dis. 2024 Jan 2;97(1):179–91. doi: 10.3233/JAD-230608 (PMC10789344; doi:10.3233/JAD-230608)
Supplement: Supplementary Material [file jad-97-jad230608-s001.pdf]

Supplementary Material

The Reliability and Clinical Validation of Automatically-Derived Verbal Memory Features of the Verbal Learning Test in Early Diagnostics of Cognitive Impairment

Supplementary Table 1. 15 Word Verbal Learning Test features

Feature name	Description
Immediate recall trial 1 – trial 5	Refers to the number of correctly recalled words of the word list for each of the 5 trials separately
Total immediate recall	Refers to the total of correctly recalled words of the word list added up from the 5 trials
Immediate repetition count trial 1 – trial 5	Refers to the number of repetitions for each of the 5 trials separately
Immediate total repetitions	Refers to the total of repetitions added up from the 5 trials
Immediate serial cluster counts trial 1 – trial 5	Measure for the reproduction of words in the same order they were presented. Calculated by dividing the number of observed serial clusters by the number of serial clusters that could be expected to occur by chance for each trial separately
Immediate total serial clusters	Refers to the total number of serial clusters summed across all learning trials
Immediate serial clustering ratio	This ratio is calculated as the total number of serial clusters summed across all learning trials divided by the total number of target words identified across all learning trials
Immediate midlist item trial 1 – trial 5	Refers to an individual recalling words in the middle (4-12) of the wordlist for each of the 5 trials separately
Immediate total midlist items	Refers to the total of recalled words in the middle (4 – 12) of the wordlist summed up for all 5 trials
Immediate primacy item trial 1 – trial 5	Refers to the recalling of the first 3 words from the word list after each of the 5 trials
Immediate total primacy items	Refers to the total of recalling of the first 3 words from the word list added up from the 5 trials
Immediate recency item trial 1 – trial 5	Refers to an individual recalling the last 3 words from the wordlist after each of the 5 trials
Immediate total recency items	Refers to the total of recalling of the last 3 words from the wordlist added up from the 5 trials
Immediate primacy index	Corresponds to [(cumulative recall of the first 3 items during trial 1 to trial 5/(total free recall for trial 1 to trial 5)] x 100
Immediate recency index	Corresponds to [(cumulative recall of the last 3 items during trial 1 to trial 5/(total free recall for trial 1 to trial 5)] x 100
Immediate constancy learning	Corresponds to [(the number of times an item correctly

index	recalled during trial 1 to trial 4 recalled during trial 5)/(total free recall for trials 1 to 4)] x 100
Immediate inter trial subjective clustering score trial 1 – trial 5	Refers to the constancies in response ordering that develop in a series of free recalls. Each time a combination of words is recalled consecutively in one trial and also in the next trial (=IRT) the subject gets one point. NOTE: serial clusters are not excluded in the computation of ITR
Immediate serial cluster relative trial 1 – trial 5	Similar to subjective clustering, but ITR only considers serial clusters (intertrial repetitions of serial clusters)
Simple learning slope	Defined as the change in recall scores between trial 1 and trial 5, divided by 4.
Regression based learning slope	Defined as the linear least squares regression of trial 1 to 5 scores on the trial numbers
Peak learning slope	Defined as the change between trial 1 recall and the earliest peak recall on trials 2 to 5, divided by the change in trial number
Late learning slope	Change in recall scores between trial 2 to trial 5
Early learning slope	Change in recall scores between trial 1 to trial 2
Correct score trial three minus trial one	Refers to the learning difference between the first and third trial
Delayed recall	Refers to total correctly recalled words in the delayed recall
Delayed recall repetitions	Refers to the total recalled repetitions in the delayed recall
Delayed recall primacy items	Refers to an individual recalling the first 3 words from the wordlist in delayed recall
Delayed recall midlist items	Refers to an individual recalling words in the middle (4-12) from the wordlist in delayed recall.
Delayed recall recency items	Refers to an individual recalling the last 3 words from the wordlist in delayed recall
Delayed recall serial clusters	Number of serial clusters for delayed recall
Delayed recall count minus highest trial correct count	Correct count in delayed recall minus the highest word count in the learning trials
Immediate trial 1 – trial 5 duration	Total duration of recall for each of the 5 trials separately
Immediate trial 1 – trial 5 mean transition time	Mean transition time between clusters of temporal clustering for each of the 5 trials separately
Immediate trial 1 – trial 5 temporal clustering mean cluster size	Mean cluster size of temporal clustering for each of the 5 trials separately
Immediate trial 1 – trial 5 temporal clustering n switches	Number of switches between temporal clusters (n_cluster - 1) for each of the 5 trials separately
Immediate trial 1 – trial 5 temporal clustering mean intra cluster transition time	Mean intra cluster transition time of temporal clustering for each of the 5 trials separately
Immediate trial 1 – trial 5 temporal clustering mean inter cluster transition time	Mean inter cluster transition time of temporal clustering for each of the 5 trials separately

Delayed recall duration	Total duration of recall for delayed recall
Delayed recall mean transition time	Mean transition time between clusters of temporal clustering for delayed recall
Delayed recall temporal clustering mean cluster size	Mean cluster size of temporal clustering for delayed recall
Delayed recall temporal clustering mean intra-cluster transition time	Mean intra cluster transition time of temporal clustering for delayed recall
Delayed recall temporal clustering mean inter cluster transition time	Mean inter cluster transition time of temporal clustering for delayed recall
Recognition count	Number of words in the learning list correctly identified on the recognition trial
False positive count	Number of words incorrectly identified on the recognition trial
True recognition	Recognition minus false positive errors
Primacy recognition	Number of words identified in the recognition trial that were from the first 1/3 of the list
Recency recognition	Number of words identified in the recognition trial that were from the final 1/3 of the list
Combination score	True recognition + primacy recognition
Recognition recall discrepancy score	Recognition score subtracted from the highest recall score

Supplementary Table 2. Post-hoc analysis of the percentage of word recall in the first 10 seconds for immediate and delayed recall.

	SCD (*N* = 69)	MCI/ dementia (*N* = 69)	p
Percentage of total words in first 10 s – trial 1 (%)	68.8 (20.0)	71.12 (28.7)	0.587
Amount of recall in first 10 s – trial 1	4.2 (1.6)	3.0 (1.5)	<0.001
Total recall trial 1	6.5 (2.8)	4.5 (2.2)	<0.001
Percentage of total words in first 10 s – trial 2 (%)	55.5 (18.5)	68.6 (23.3)	<0.001
Amount of recall in first 10 s – trial 2	4.9 (1.7)	4.3 (1.7)	0.033
Total recall trial 2	9.3 (3.0)	6.6 (2.9)	<0.001
Percentage of total words in first 10 s – trial 3 (%)	54.7 (19.1)	65.7 (23.1)	0.003
Amount of recall in first 10 s – trial 3	5.9 (2.0)	4.5 (1.7)	<0.001
Total recall trial 3	11.0 (2.7)	7.22 (2.7)	<0.001
Percentage of total words in first 10 s – trial 4 (%)	54.4 (19.9)	63.5 (24.1)	0.017
Amount of recall in first 10 s – trial 4	6.0 (2.2)	4.7 (1.8)	<0.001
Total recall trial 4	11.6 (3.0)	8.2 (3.1)	<0.001
Percentage of total words in first 10 s – trial 5 (%)	48.6 (18.8)	61.4 (24.6)	0.001
Amount of recall in first 10 s – trial 5	5.8 (2.0)	4.8 (2.0)	0.003
Total recall trial 5	12.5 (2.5)	8.5 (3.6)	<0.001
Percentage of total words in first 10 s – delayed recall (%)	49.1 (19.1)	52.11 (26.9)	0.471
Amount of recall in first 10 s – delayed recall	5.2 (2.4)	2.7 (2.1)	<0.001
Total delayed recall	10.8 (3.5)	5.7 (4.2)	<0.001

SCD, subjective cognitive decline; MCI, mild cognitive impairment
